# The Detection and Reactivity of Silanols and Silanes Using Hyperpolarized ^29^Si Nuclear Magnetic Resonance

**DOI:** 10.1002/anie.201915098

**Published:** 2020-01-09

**Authors:** Peter J. Rayner, Peter M. Richardson, Simon B. Duckett

**Affiliations:** ^1^ Centre of Hyperpolarisation in Magnetic Resonance Department of Chemistry University of York Heslington YO10 5DD UK

**Keywords:** catalysis, hyperpolarization, NMR spectroscopy, SABRE, silicon

## Abstract

Silanols and silanes are key precursors and intermediates for the synthesis of silicon‐based materials. While their characterization and quantification by ^29^Si NMR spectroscopy has received significant attention, it is a technique that is limited by the low natural abundance of ^29^Si and its low sensitivity. Here, we describe a method using p‐H_2_ to hyperpolarize ^29^Si. The observed signal enhancements, approaching 3000‐fold at 11.7 T, would take many days of measurement for comparable results under Boltzmann conditions. The resulting signals were exploited to monitor the rapid reaction of tris(*tert*‐butoxy)silanol with triflic anhydride in a *T*_1_‐corrected process that allows for rapid quantification. These results demonstrate a novel route to quantify dynamic processes and intermediates in the synthesis of silicon materials.

## Graphical abstract

**The hyperpolarization** of silanols and silanes was achieved using parahydrogen and an iridium catalyst. The resulting ^29^Si NMR signal gains approach a factor of 3000 and were harnessed for the quantitative monitoring of a substitution reaction.

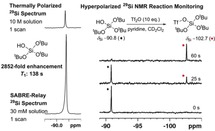

Silicon is one of the most abundant elements known to man. Silicon‐based materials find use in a wide range of applications, from the synthesis of bulk materials through to roles in molecular transformations. Of its three naturally occurring isotopes, only ^29^Si has a non‐zero magnetic moment, and its nuclear magnetic resonance (NMR) detection is of wide interest. Its wide chemical shift dispersion makes it highly diagnostic for characterization purposes and allows the examination of dynamic processes in solution and the solid state.^1^ However, ^29^Si NMR spectroscopy has limitations that reduce its potential use,^2^ primarily due to its low sensitivity that is dependent upon the small population differences that exist between nuclear spin energy levels within a magnetic field. These populations are governed by the Boltzmann distribution, and therefore, a ^29^Si NMR signal reflects just 1 in 125 500 of these nuclei at 11.7 T. Its 4.7 % natural abundance and typically long *T*_1_ values also hinder detection.^1^ Furthermore, as it is found in materials that make up an NMR probe, and an NMR tube, the broad background signal can impede the detection of low‐concentration species.

Hyperpolarization is a route to overcome this insensitivity and refers to a situation whereby the spin energy level populations are perturbed from Boltzmann equilibrium conditions. These techniques have been utilized in both medical and analytical science, and with respect to ^29^Si, a number of hyperpolarized magnetic resonance applications using dynamic nuclear polarization (DNP) have been reported.^3^ The parahydrogen (p‐H_2_)^4^ based method, signal amplification of reversible exchange (SABRE),^5^ has been applied to the polarization of ^1^H and a number of heteronuclei, including ^29^Si.^6^ However, these studies were limited to N‐heterocyclic functionalized silanes because of the need to bind the substrate to the transition‐metal polarization transfer catalyst.

The SABRE‐Relay technique^7^ overcomes this barrier, and consequently, the scope of p‐H_2_‐based hyperpolarization has been greatly expanded.^8^ Now, a carrier agent, such as an amine, becomes hyperpolarized through the initial formation of an active polarization transfer catalyst of the type [Ir(H)_2_(NHC)(RNH_2_)_3_]Cl as depicted in Scheme 1. The hyperpolarized carrier amine enhances the target substrate via proton transfer. Here, we develop the SABRE‐Relay technique for the hyperpolarization of silanols by p‐H_2_ and direct silane polarization by SABRE.

**Scheme 1:**
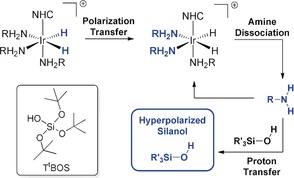
SABRE‐Relay polarization transfer proceeds through the scalar coupling network between the p‐H_2_‐derived hydride ligands into the carrier amine (RNH_2_). Ligand dissociation and proton transfer yield a spin‐hyperpolarized silanol (R′_3_SiOH). Inset: Tris(*tert*‐butoxy)silanol (T^t^BOS).

The Stöber process^9^ is the method of choice for the synthesis of silica‐based materials. It is a sol–gel process^10^ that begins with the hydrolysis of a tetra orthosilicate to give a mixture of silanols, which are subsequently condensed to form the material. The nature of the silanol intermediates determines the physical properties of these materials,^11^ and therefore, methods for their rapid and cost‐efficient detection and characterization are desirable. Our starting point was to take tris(*tert*‐butoxy)silanol (T^t^BOS; inset, Scheme 1) as a model substrate to develop the p‐H_2_‐derived hyperpolarization of silanols. A 5 mm NMR tube fitted with a J. Young's tap containing a solution of [IrCl(COD)(IMes)] (**1**, 5 mm), benzylamine‐*d*_7_ (BnNH_2_‐*d*_7_, 50 mm), and T^t^BOS (50 mm) in CD_2_Cl_2_ (0.6 mL) was placed under a 3 bar atmosphere of H_2_. After 1 h at 298 K, the resulting ^1^H NMR spectrum showed clean conversion into [Ir(H)_2_(IMes)(BnNH_2_ *‐d*_7_)_3_]Cl.^7^, ^8^ No evidence for T^t^BOS binding to this iridium center was observed by NMR or MS methods. After shaking this sample under 3 bar p‐H_2_ for 10 s at 298 K at 70 G, it was rapidly transferred into an 11.7 T spectrometer for interrogation by ^29^Si NMR spectroscopy. A ^29^Si signal that was 82±5‐fold more intense than that in the corresponding thermally equilibrated control spectrum was detected. The transfer of hyperpolarization from the silanol ^1^H to the ^29^Si is likely to occur through both low‐field thermal mixing^12^ and nuclear Overhauser enhancement.^13^ Consequently, we sought to improve the ^29^Si NMR signal gain to enable in situ reaction monitoring.

We began by varying the identity of the catalyst's N‐heterocyclic carbene (NHC) ligand as it has been shown to affect the observed polarization level. The selective ^2^H labeling of the NHC can result in increased levels of polarization because of reduced spin dilution and longer *T*_1_ relaxation times.^14^ Therefore, an analogous SABRE‐Relay sample was prepared using ***d***_**22**_ **‐1**. After SABRE‐Relay transfer, an improved silanol signal gain of 92±4‐fold was observed (Figure 1 B). Modification of the steric and electronic properties of the NHC ligand has also been shown to modulate the ligand dissociation rates and thereby lead to better SABRE enhancement.^15^ The rate of equatorial BnNH_2_ dissociation from [Ir(H)_2_(IMes)(BnNH_2_ *‐d*_7_)_3_]Cl is 3.33 s^−1^ and therefore lower than predicted to be optimal.^16^ Bulky catalyst **2**, furnished with *tert*‐butyl groups, increases the signal gain to 128±11‐fold whereas its isotopologue, ***d***_**34**_ **‐2**, gave a 150±9‐fold gain. Catalyst **3**, which bears the even more bulky SIMes ligand, also improved the ^29^Si signal gain compared to **1**. Continuing with this trend, we were able to further increase the steric effects of the NHC through the use of catalysts **4** (IPr) and **5** (SIPr), which gave signal enhancements of 157±15 and 310±22, respectively. We would expect these signal enhancements to be further improved by the use of the deuterated isotopologues and are currently exploring routes to their synthesis.

**Figure 1:**
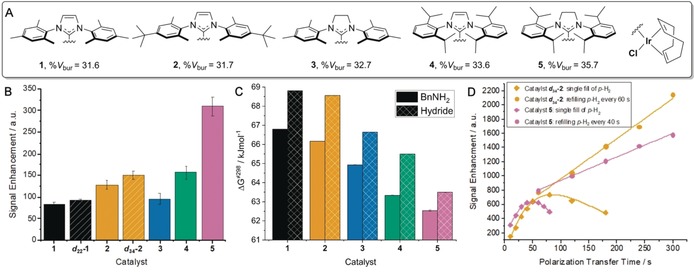
Catalyst effects on the SABRE‐Relay polarization of T^t^BOS. A) Structures of catalysts and their buried volumes (% *V*_bur_).^15^ B) SABRE‐Relay signal enhancements using [IrCl(COD)(NHC)] (5 mm), BnNH_2_‐*d*_7_ (50 mm), and T^t^BOS (50 mm) in CD_2_Cl_2_ (0.6 mL), 3 bar p‐H_2_ for 10 s at 70 G. C) Δ*G* ^≠298^ values for BnNH_2_ (solid) and hydride (hashed) ligand dissociation from the active catalysts of type [Ir(H)_2_(NHC)(BnNH_2_)_3_]Cl. D) Polarization transfer time effect on signal enhancement of T^t^BOS when using ***d***_**34**_ **‐4** (orange) and **5** (pink) with a single filling of p‐H_2_ (diamonds) or multiple refills (squares).

In order to rationalize these differences in polarization level, we calculated the Δ*G* ^≠298^ value of ligand dissociation for equatorially bound BnNH_2_ and H_2_ loss for the active complexes. These values were calculated as described in the Supporting Information. The barrier to H_2_ loss is consistently higher than that of BnNH_2_ dissociation for each catalyst, which is consistent with the expected dissociative mechanism (Figure 1 C). The catalyst derived from **1**, [Ir(H)_2_(IMes)(BnNH_2_)_3_]Cl, gave Δ*G* ^≠298^ values of 66.79 and 68.81 kJ mol^−1^ for BnNH_2_ and H_2_ loss, respectively. These Δ*G* ^≠298^ values decrease when the steric bulk of the NHC ligand is increased across the series of catalysts **1**–**5**. For catalyst **2**, they are 66.16 and 68.56 kJ mol^−1^, respectively. Catalyst **5**, which gave the largest ^29^Si signal enhancements after 10 s polarization transfer, has the lowest values of Δ*G* ^≠298^ (62.54 and 63.5 kJ mol^−1^ for BnH_2_ and H_2_ loss, respectively). These data confirm that lower barriers to ligand loss promote more effective SABRE‐Relay transfer. This effect is likely to be attenuated by relaxation of the NH protons of BnNH_2_‐*d*_7_ in the presence of [Ir(H)_2_(SIPr)(BnNH_2_‐*d*_7_)_3_]Cl whose *T*_1_ was now just 0.8 s at 11.7 T. Thus, rapid ligand exchange allows for the effective replenishment of the polarized transfer agent; however, if this exchange is too fast, rapid relaxation will limit hyperpolarization buildup whilst depleting the p‐H_2_.

The corresponding hyperpolarized ^29^Si signal lifetime of T^t^BOS was 138.4 s as measured by a variable flip angle pulse sequence (see the Supporting Information). As the decay of the created ^29^Si magnetization is slow, we postulated that the NMR signal gains could be increased if the SABRE‐Relay time was extended beyond 10 s. Consequently, an increase in signal gain to 625±34‐fold was obtained using **5** when the polarization time was extended to 50 s (Figure 1 D). Extending this time further decreased the signal intensity because of the finite amount of p‐H_2_ becoming limiting during the SABRE‐Relay process. Upon repeating this experiment with ***d***_**34**_ **‐2**, a signal gain of 767±38‐fold was reached after 70 s exposure to p‐H_2_. We conclude that while **5** leads to a more rapid buildup of polarization, its higher rate of ligand exchange consumes the p‐H_2_ in the sample. Complex ***d***_**34**_ **‐2** yields higher signal gains with extended polarization transfer times and the same finite volume of p‐H_2_ as relaxation effects are reduced by slower exchange. In support of this, the relaxation times of the NH protons of BnNH_2_‐*d*_7_ in the presence of ***d***_**34**_ **‐2** were measured to be 3.2 s at 11.7 T.

The polarization times were extended further by evacuating and refilling the NMR tubes containing the SABRE‐Relay solutions with p‐H_2_ periodically until 300 s was reached. For **5**, the time between fills was 40 s, and for ***d***_**34**_ **‐2**, it was 60 s. For both catalysts, a linear increase in ^29^Si polarization level was observed over time but ***d***_**34**_ **‐2** led to the highest signal gain of 2142±180‐fold after 300 s. For **5**, the signal gain was 1580±120‐fold. This behavior is reflected in the lower magnetization buildup slope illustrated in Figure 1 D. Increasing the pressure of p‐H_2_ from 3 bar to 5 bar yielded a further 10 % increase in signal gain to 2313‐fold with ***d***_**34**_ **‐2** due to increased p‐H_2_ availability. An automated polarizer, which introduces a constant flow of p‐H_2_ into the solution, gave a similar linear increase in signal gain; however, the signal gains only reach a maximum of 100‐fold (see the Supporting Information). We suggest that this is due to inefficient mixing of p‐H_2_ in solution.

After optimization, we concluded that the best conditions for the polarization T^t^BOS are ***d***_**34**_ **‐2** (5 mm), BnNH_2_‐*d*_7_ (50 mm), and T^t^BOS (30 mm) in CD_2_Cl_2_ (0.6 mL) and exposure to p‐H_2_ at 5 bar for 300 s at 70 G with refreshing the p‐H_2_ atmosphere every 60 s. This yields a total signal gain of 2852±112‐fold (2.3 %) in the ^29^Si NMR spectrum (Figure 2). These conditions were then applied to the polarization of a number of other silanols as summarized in Figure 2. As their *T*_1_ relaxation times are shorter than that of T^t^BOS, the maximum signal gains are achieved with shorter total polarization transfer times.

**Figure 2:**
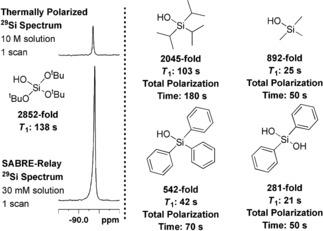
Silanol ^29^Si NMR response results after hyperpolarization by SABRE‐Relay transfer using ***d***_**34**_ **‐2** (5 mm), silanol (30 mm), and BnNH_2_‐*d*_7_ (50 mm) in CD_2_Cl_2_ (0.6 mL) at 5 bar p‐H_2_.

DNP has previously been used for quantitative reaction rate determination using ^1^H and ^13^C nuclei.^17^ The hyperpolarized ^29^Si NMR signals and the long *T*_1_ relaxation time of T^t^BOS were exploited to monitor a molecular transformation. The nucleophilic substitution of triflic anhydride (Tf_2_O) with T^t^BOS was chosen as it has been reported as a method for functionalization of silica surfaces.^18^ A relaxation‐corrected variable flip angle sequence was used to overcome the loss of magnetization due to *T*_1_ relaxation during the reaction and to give an immediate concentration profile as detailed in the Supporting Information.^19^ The SABRE‐Relay polarization of T^t^BOS was conducted using our previously optimized conditions, prior to the introduction of a solution of Tf_2_O (10 equiv) and pyridine (10 equiv) in CD_2_Cl_2_ (0.1 mL). Subsequent rapid sample insertion into an 11.7 T magnetic field prior to acquisition of a ^29^Si spectrum every 5 s for a duration of 60 s (Figure 3). When Tf_2_O was present in excess, we observed conversion of T^t^BOS (*δ*_Si_=−90.8 ppm) into its triflate derivative (*δ*_Si_=−102.7 ppm). The identity of these signals was unequivocally confirmed by independent synthesis (see the Supporting Information). After an induction period of 10 s, which we attribute to diffusion of the Tf_2_O into the NMR detection region, the expected pseudo‐first‐order consumption of the starting silanol and the corresponding production of its triflate product was observed. The rate constant for this was determined to be 0.070±0.001 s^−1^ and is not affected by changing the concentration of the SABRE catalyst, which confirms that the catalyst does not participate in the nucleophilic reaction. This data would not be possible to collect using ^29^Si NMR spectroscopy under Boltzmann conditions because of the requirement for signal averaging and long *T*_1_ values; the speed of this reaction means that it would be complete before the first measurement could be made. When the reaction was repeated with substoichiometric quantities of Tf_2_O, a new resonance at *δ*_Si_=−93.2 ppm was observed, which we attribute to the product of dimerization (see the Supporting Information). It is formed by reaction of T^t^BOS with its triflate intermediate in a two‐step process. The same signal is present in the ^29^Si NMR spectrum when the reaction was repeated with tris(*tert*‐butoxy)silyl chloride; however, the reaction is now too rapid to derive any kinetic data. As the oligomerization of silanols is a key step in the synthesis of silica materials, the result demonstrates that it may be possible to detect and quantify intermediates in the sol–gel process.

**Figure 3:**
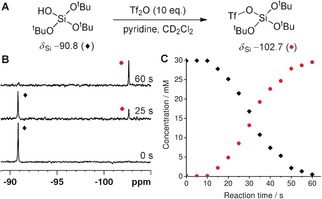
Hyperpolarized nucleophilic substitution of Tf_2_O with T^t^BOS. A) Reaction scheme. B) SABRE‐Relay hyperpolarized ^29^Si spectra at indicated reaction time points. C) Concentration of T^t^BOS (⧫) and the triflate product (•) as a function of reaction time calculated from the corresponding *T*_1_‐corrected hyperpolarized ^29^Si NMR spectra.

During the course of our investigation into hyperpolarized ^29^Si NMR spectroscopy, we also discovered a method to hyperpolarize important silanes via SABRE. When a sample containing ***d***_**22**_ **‐1** (5 mm), dimethylethoxysilane (50 mm), and BnNH_2_‐*d*_7_ (50 mm) in CD_2_Cl_2_ (0.6 mL) was shaken at 70 G for 10 s, a hyperpolarized signal gain of 206±24‐fold was observed in the ^29^Si NMR spectrum as an antiphase doublet (*J*_Si–H_=205 Hz; Figure 4 B). Antiphase character is typically seen for two inequivalent p‐H_2_‐derived hydride ligands;4b, 5c, 12 here, similarly complex polarization is spontaneously created, but now shared between a ^29^Si and a ^1^H nucleus. The *T*_1_ value for this signal was measured to be 38±1.2 s. In the ^1^H NMR spectrum, after SABRE polarization, the intensity of the Si−H resonance is 70±5 times greater than in a thermally equilibrated reference spectrum. The dominant hydride‐containing species in the ^1^H NMR spectrum was [Ir(H)_2_(*d*_22_‐IMes)(BnNH_2_)_3_]Cl,8a and no hydride ligands were seen that could be attributed to a silane complex. As SABRE transfer is seen when a 70 G field is employed, it demonstrates the existence of a polarization transfer route involving p‐H_2_‐derived hydride ligands, and dihydride‐η^2^‐silane complexes have been exemplified elsewhere.^20^ We searched spectroscopically for the presence of such a species at low temperature in our SABRE sample; however, no signals attributable to an intermediate η^2^‐silane complex could be detected.

**Figure 4:**
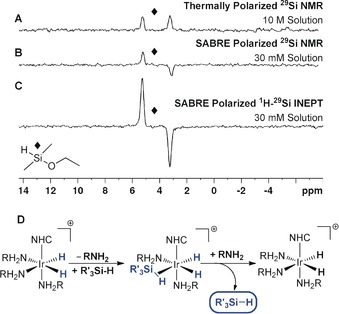
SABRE hyperpolarization of dimethylethoxysilane. A) Thermally polarized ^29^Si NMR spectrum of a 10 m solution. B) ^29^Si NMR spectrum after SABRE hyperpolarization with [IrCl(COD)(*d*_22_‐IMes)] (***d***_**22**_ **‐1**) and 3 bar p‐H_2_ at 70 G. C) ^1^H–^29^Si INEPT NMR spectrum after SABRE hyperpolarization under analogous conditions. D) Proposed route for silane hyperpolarization.

After SABRE catalysis at 70 G, a ^1^H–^29^Si INEPT based transfer sequence was utilized at 11.7 T to create the same antiphase signal as that of Figure 4 B with an increased signal gain of 772±56 (Figure 4 C). The use of alternative co‐ligands, such as DMSO‐*d*_6_ and CD_3_CN, did not increase the signal gains when compared to those with BnNH_2_‐*d*_7_. Additionally, when the sample is placed under a D_2_ atmosphere (3 bar) at room temperature for 24 h, a 60 % reduction in the Si−H signal was observed in the ^1^H NMR spectrum, thus indicating that while H/D exchange occurs, it is slow. No Si−H site exchange was observed by EXSY methods on the timescale of relaxation, which further suggests that the key intermediate is of an η^2^‐silane type rather than an oxidative silyl hydride, which would undergo rapid Si−H scrambling.

Changing the silane to pentamethyldisiloxane gave a ^29^Si signal gain of 252±22 in the ^1^H–^29^Si INEPT spectrum. However, a number of other silanes, such as triethoxysilane and triphenylsilane, yielded no SABRE catalysis and therefore highlight the sensitivity of this approach to the steric and electronic properties of the silane. Work is now ongoing to further characterize the intermediates involved in this process in order to broaden scope and applicability.

In summary, we have demonstrated the hyperpolarization of silanols and silanes using p‐H_2_. Development of the SABRE‐Relay method^7^ gave large ^29^Si signal gains that approach 3000‐fold. The effects of catalyst structure and their influence on ligand exchange processes were determined through calculation of Δ*G* ^≠298^ values and the influence on carrier amine *T*_1_ relaxation rates. The large polarization levels attained and the long *T*_1_ values were exploited using a relaxation‐corrected variable flip angle pulse sequence to measure kinetic data for the reaction of a silanol and Tf_2_O through a ^29^Si NMR response. Finally, silanes have been shown to be amenable to SABRE‐based polarization transfer when BnNH_2_‐*d*_7_ was used as a co‐ligand to give ^29^Si signal gains of 772±56‐fold. We propose that an iridium dihydride‐η^2^‐silane is the active catalyst in this process,^20^ and full rationalization of this SABRE pathway is ongoing. We anticipate that the hyperpolarized methods exemplified here for ^29^Si will extend more generally to other slow‐relaxing heteronuclei such that intermediate characterization by NMR spectroscopy is widely supported.

## Conflict of interest

The authors declare no conflict of interest.

## Supporting information

As a service to our authors and readers, this journal provides supporting information supplied by the authors. Such materials are peer reviewed and may be re‐organized for online delivery, but are not copy‐edited or typeset. Technical support issues arising from supporting information (other than missing files) should be addressed to the authors.

Supplementary
