# Supplementary material for: The Detection and Reactivity of Silanols and Silanes Using Hyperpolarized 29Si Nuclear Magnetic Resonance
Source: Angew Chem Int Ed Engl. 2020 Jan 9;59(7):2710–4. doi: 10.1002/anie.201915098 (PMC7027454; doi:10.1002/anie.201915098)
Supplement: Supplementary file 1 — Supplementary [file ANIE-59-2710-s001.pdf]

Angewandte Chemie
Eine Zeitschrift der Gesellschaft Deutscher Chemiker

Supporting Information

The Detection and Reactivity of Silanols and Silanes Using Hyperpolarized ^{29}Si Nuclear Magnetic Resonance

*Peter J. Rayner, Peter M. Richardson, and Simon B. Duckett**

anie_201915098_sm_miscellaneous_information.pdf

Supporting Information

**1 NMR polarization transfer experiments**	**S2**
**2 Synthesis and Characterisation of Silanols**	**S4**
**3 T_1 Corrected Single-Shot Sequence**	**S4**
**4 Dimerization of tris(*tert*-butoxy)silanol**	**S10**
**5 Calculation of Gibbs Free Energy**	**S11**
**5 References**	**S12**

1 NMR polarization transfer experiments

1.1 SABRE-Relay polarization transfer method

A solution containing [IrCl(COD)(NHC)] (5 mM), an amine and the indicated silanol at the specified loading in dichloromethane-d_2 (0.6 mL) was placed into an NMR tube

equipped with a J. Youngs valve. The sample was degassed using a freeze-pump-thaw method prior to the introduction of *para*hydrogen at a pressure of 3 bar. Samples were then shaken for 10 s at the specified magnetic field before being rapidly transported into the magnet for subsequent interrogation by NMR spectroscopy.

1.2 NMR Spectrometer

NMR spectra were typically acquired on a 500 MHz Bruker, Avance III console using a 5 mm BBO probe which was tuned to ^{1}H or ^{29}Si as specified. Resonances are referenced relative to the residual proton signal of the indicated deuterated solvent or tetramethylsilane where appropriate.

1.3 Determination of Enhancement factors

^{1}H NMR enhancement factors were calculated by comparison to single scan reference spectra of the corresponding sample after thermal equilibration in the magnet field. ^{29}Si NMR enhancement factors were calculated by comparison to a single scan reference spectrum high concentration standard sample after thermally equilibration in the magnetic field and scaled according to literature methods.[1]

1.4 Effect of Silanol Equivalents on SABRE-Relay Enhancement

The effect of the concentration of tris(*tert*-butoxy)silanol on the signal enhancement was probed from 10-50 mM. The conditions used for SABRE-Relay were: [IrCl(COD)(IMes)] (5 mM), d_7-BnNH$_2$ (50 mM) in dichlormethane-d_2 (0.6 mL) with 3 bar p-H$_2$ and shaking at 70 G for 10 seconds. The results are shown in Figure 1a. The highest signal enhancement of 111±15-fold was obtained at 10 mM of tris(*tert*-butoxy)silanol and a similar enhancement of 102±5-fold was also seen at 30 mM concentration. However, the absolute signal intensity was higher for the 30 mM sample than for the 10 mM sample as shown in Figure 1b. For applications where the absolute signal is of more importance than the signal enhancement, then increasing the concentration of tris(*tert*-butoxy)silanol can be beneficial.

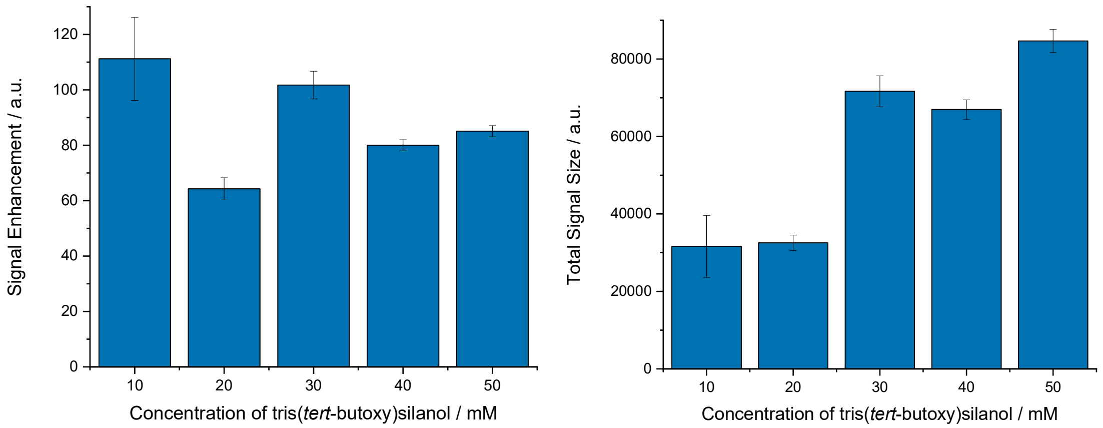

Figure S1: Effect of varying reagent concentration on the signal enhancement of tris(*tert*-butoxy)silanol when using ***d*$_{34}$-2** (5 mM) as the catalyst and *d*$_7$-BnNH$_2$ (50 mM) as the carrier agent.

1.5 Effect of Polarization Transfer Field

The field at which polarization transfer was conducted was varied using static magnetic fields from 30-140 G. Polarization transfer under SABRE-Relay was conducted for 30 seconds on a sample containing ***d*$_{34}$-2** (5 mM), tris(*tert*-butoxy)silanol (50 mM), *d*$_7$-BnNH$_2$ (50 mM) and 3 bar *p*-H$_2$ in dichloromethane-*d*$_2$. Figure S2 shows that the largest signal enhancements were observed when this process was undertaken between 60 and 80 G. This is the same field wherein the amine receives the strongest SABRE hyperpolarization.

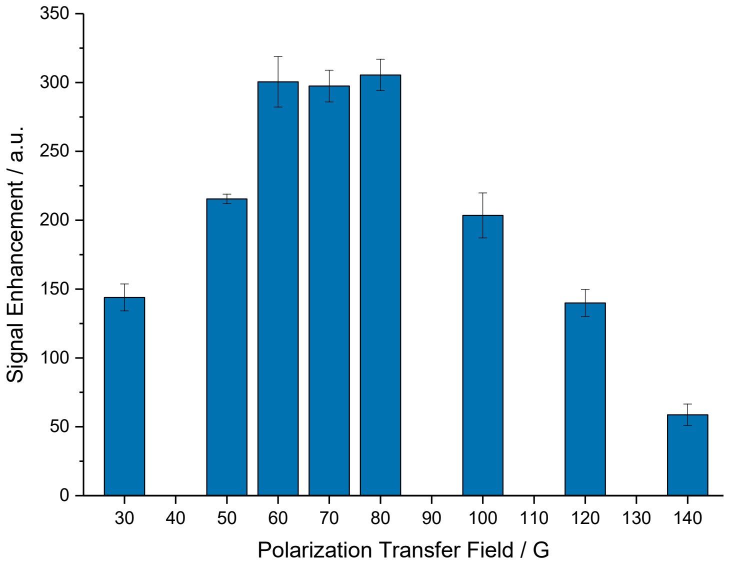

Figure S2: Effect of varying the polarization transfer field on the ^{29}Si NMR signal enhancement seen for tris(*tert*-butoxy)silanol when using ***d*$_{34}$-2** (5 mM) as the catalyst and *d*$_7$-BnNH$_2$ (50 mM) as the carrier agent under 3 bar *p*-H$_2$.

1.6 Use of an Automated Polarization Transfer Device

An automated polarizer[2] was utilized for the SABRE-Relay hyperpolarization of tris(*tert*-butoxy)silanol (50 mM) using ***d*₃₄-2** (5 mM) as the catalyst and d_7-BnNH$_2$ (50 mM) as the transfer agent. The time of bubbling *p*-H$_2$ through the solution was varied between 30 and 240 seconds. Figure S3 shows the effect of bubbling time on the signal enhancement measured at 11.7 T.A linear increase in signal enhancement is observed over the time period however the total signal gain is significantly lower than that achieved with the manual shaking method.

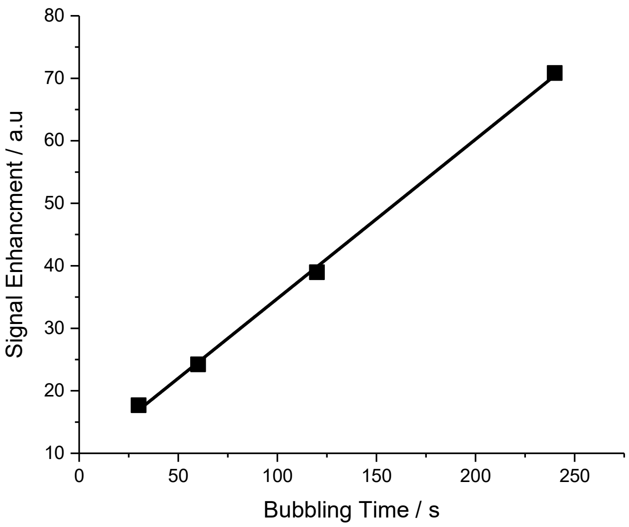

Figure S3: Effect of varying the bubbling times on the ^{29}Si NMR signal enhancement seen for tris(*tert*-butoxy)silanol when using $\boldsymbol{d_{34}}$**-2** (5 mM) as the catalyst and d_7-BnNH$_2$ (50 mM) as the carrier agent in conjunction with an automated polarizer.

2 Synthesis and Characterisation of Silanols

2.1 Tris(*tert*-butoxy)silanol

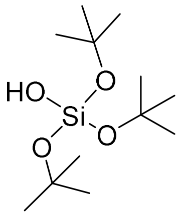

Tris(*tert*-butoxy)silanol was purchased from Merck (CAS:18166-43-3, SKU: 553468) and used without further purification. ^{1}H NMR (500 MHz, CD$_2$Cl$_2$) δ 3.30 (s, 1H, OH), 1.37 (s, 27 H, CH$_3$); ^{13}C NMR (125 MHz, CD$_2$Cl$_2$) δ 72.82 (s, <u>C</u>CH$_3$), 31.05 (CH$_3$); ^{29}Si NMR (99 MHz, CD$_2$Cl$_2$) δ −90.66 (s).

2.2 Tri(*tert*-butoxy)silyl triflate

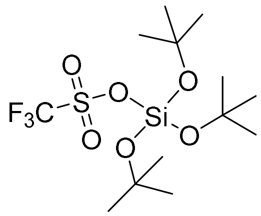

Triflic anhydride (18.6 µL, 0.11 mmol) was added to a stirred solution of tris(tert-butoxy)silanol (26.4 mg, 0.1 mmol) in dichloromethane-d_2 (0.6 mL) at 0 °C. The resulting solution was warmed to room temperature for 5 minutes and examined by NMR which showed ca. 85% conversion into the product. ^{1}H NMR (500 MHz, CD_2Cl_2) δ 1.41 (s, 27 H, CH_3); ^{13}C NMR (125 MHz, CD_2Cl_2) δ 115.32 (q, J = 284 Hz, CF_3), 76.24 (s, <u>C</u>CH_3), 31.46 (CH_3); ^{29}Si NMR (99 MHz, CD_2Cl_2) δ −102.68 (s).

3 T_1 Corrected Single-Shot Sequence

The variable pulse duration sequence of Figure S4 was used to measure hyperpolarised T_1 values of the ^{29}Si centre and shown to work effectively. This sequence employs a train of pulse and acquire style segments that are separated by a fixed delay to encode relaxation. The initial pulse angle is selected to provide sufficient signal to measure a response without using a significant amount of the available polarisation. In previous work from the group an angle of around 15° proved suitable for ^{1}H detection as a first pulse which gives an overall signal of around $0.25 \times M_z$, where M_z is the maximum available magnetisation derived from the hyperpolarised sample at time zero. Each subsequent pulse has a longer duration, and hence, flip angle, with the intention to sample the sample amount of the available magnetisation at each point. However, the available polarisation will also be undergoing T_1 relaxation throughout this process. Therefore, by sampling the same amount of polarisation neglecting the T_1 relaxation for each point and measuring the amount of resulting detectable magnetisation a value of T_1 can be determined. This has recently been shown to work well for measuring hyperpolarisation lifetimes using SABRE.[3]

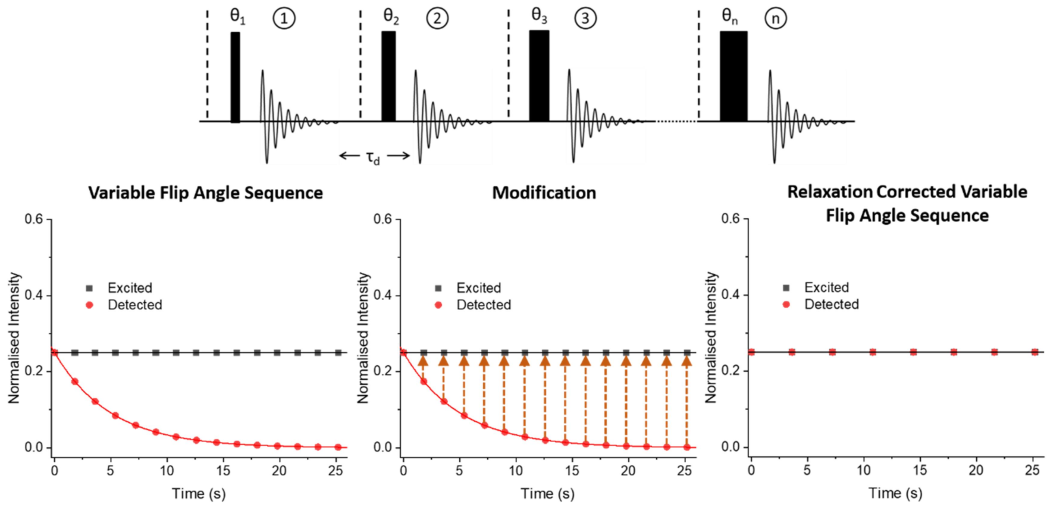

Figure S4 – Schematic of the applied pulse sequence which is applied multiple times with increasing angle with each subsequent pulse. Left graph shows schematic of the amount of theoretical excited magnetisation in the absence of T_1 and then the detected signal due only to relaxation. Middle graph highlights the projected changes utilised here to allow the theoretically excited and detected to be the same which is shown in the right hand side graph.

The reaction monitoring pulse sequence will then detect an amount of magnetisation that is governed by the T_1 value and any preceding excitations (schematic shown in Figure S4). In order to sample the same amount of polarisation each time, and consequently turn the measurement into a quantitative response, longer pulse angles are needed as the experiment duration increases. Consequently, the magnetisation left along the z-axis ($M_{z,n}$) at any point in the experiment can be defined as the amount of magnetisation present before the previous pulse ($M_{z,n-1}$) after applying a reduction factor based on applying the previous pulse ($cos(\vartheta_{n-1})$) and the exponential reduction that arises from relaxation between the current time (t_n) and previously sampled time (t_{n-1}), where n is the pulse number starting at 1 and T_1 is the hyperpolarisation lifetime (equation 1).

$$M_{z,n} = M_{z,n-1}.cos(\vartheta_{n-1}).e^{-(t_n - t_{n-1})/T_1} \quad (1)$$

Now that the magnetisation along the z-axis is defined immediately before any pulse in the experiment the detectable magnetisation in the xy-plane ($M_{xy,n}$) can be readily defined in equation 2 where ϑ_n is the angle used at point n.

$$M_{xy,n} = M_{z,n}.\sin(\vartheta_n) \quad (2)$$

In order to measure the same amount of magnetisation in the xy-plane with each pulse, the value of $M_{xy,n}$ must remain constant throughout the experiment. By simple rearrangement of equation 2 the required angle ϑ_n can be found as in equation 3.

$$\vartheta_n = \sin^{-1}\left(\frac{M_{xy,n}}{M_{z,n}}\right) \quad (3)$$

Therefore, if equation 1 is used to determine the value of $M_{z,n}$ and $M_{xy,n}$ is set as constant then a list of angles can be chosen to provide a constant level of detected magnetisation throughout the experiment. There will be a clear trade off here between the amount of signal acquired and both the number of detectable points and the time between points (due to relaxation of the magnetisation). A variable pulse duration ($v_{p,n}$) list can be easily determined if an accurate duration of a standard 90° (d_{90}) is carefully calibrated, by using equation 4.

$$v_{p,n} = \frac{\vartheta_n}{90} . d_{90} \quad (4)$$

This sequence has been tested with ^{15}N hyperpolarisation initially as shaking in mG fields provides significant enhancement on ^{15}N enriched pyridine under standard SABRE conditions. ^{15}N was chosen as a test candidate as this sequence will only work with nuclei which will provide a negligible thermal response for example ^{15}N, ^{13}C and ^{29}Si. It should be noted that from the formulation above the parameters $M_{z,0}$ has an inherent value of 1 and t_0 and ϑ_n having the initial value of 0.

Table 1 shows the values calculated for a sample containing 5.2 mM [IrCl(COD)(*d*$_{22}$-IMes)] with 52 mM of ^{15}N labelled pyridine in methanol-*d*$_4$. The T_1 value for this sample was determined as 29.0 s using the single-shot method described above. For this reason, the time points were chosen to go just beyond one T_1 and thus 10 points were sampled with 5 seconds between each acquisition. Care needs to be taken to include all sequence timings, including pulse durations and inherent writing data times in the software. Equation 1 was used to calculate the values of $M_{z,n}$ which has been normalised to start at a value of 1. $M_{xy,n}$ was then iterated until 10 points were achieved with minimal signal left over after the experiment. It can be seen that after the final point has been acquired there should only be 1.3 % of the signal wasted. A value of 0.116 was used for $M_{xy,n}$ which translates to observing 11.6 % of the total available hyperpolarised signal every point, which is sufficient since the level of hyperpolarisation is significant. ϑ_n and $v_{p,n}$ have been determined using equations 3 and 4 respectively.

The 11th point has been included in Table S1 to highlight that there is insufficient polarisation left to sample another point. The number of available points to sample is dependent on the T_1 and the time between points (Δt_n). The d_{90} used for this sample was 27 μs which has been calibrated from a standard of a different system. The accuracy of d_{90} and T_1 are important must be carefully considered.

n	t_n(s)	$M_{z,n}$	$M_{xy,n}$	ϑ_n (°)	$v_{p,n}$ (µs)	I_{norm} (%)
1	0	1.000	0.116	6.66	2.00	100.0
2	5	0.836	0.116	7.98	2.39	99.1
3	10	0.697	0.116	9.58	2.88	96.8
4	15	0.578	0.116	11.57	3.47	96.2
5	20	0.477	0.116	14.08	4.23	95.7
6	25	0.389	0.116	17.34	5.20	94.8
7	30	0.313	0.116	21.78	6.53	95.9
8	35	0.244	0.116	28.34	8.50	96.6
9	40	0.181	0.116	39.85	11.96	97.6
10	45	0.117	0.116	82.63	24.79	108.1
11	50	0.013	0.116	N/A	N/A	N/A

Table S1 – Calculated parameters for a sample containing 5.2 mM [IrCl(COD)(d_{22}-IMes)] with 52 mM of ^{15}N labelled pyridine in methanol-d_4, which has a measured T_1 of 29.0 s and d_{90} of 27 µs. All parameters were calculated from equations 1, 3 and 4 with the exception of the I_{norm} parameter, which is the normalised integrals of the experimental data using listed flip angles.

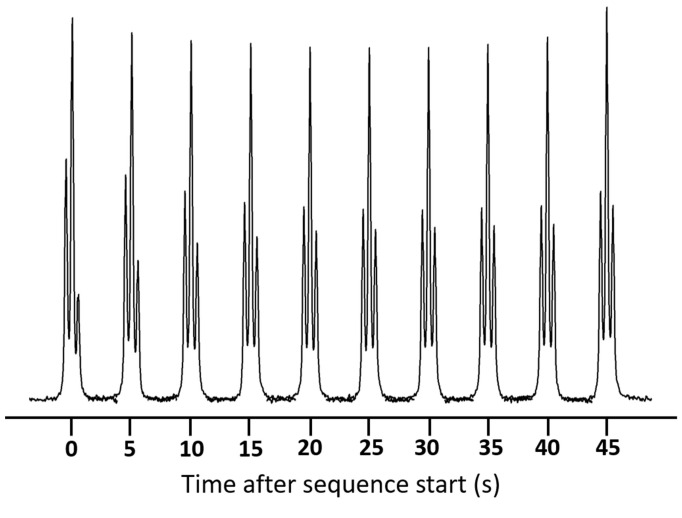

Figure S5 – Spectra measured as a function of time measured after initial pulse, using the parameters listed in Table 1 for a sample containing 5.2 mM [IrCl(COD)(d_{22}-IMes)] with 52 mM of ^{15}N labelled pyridine in methanol-d_4. The sample was loaded with 4 Bar of p-H_2 (99%) and shaken for 10 s in a 5 mG magnetic field obtained using a magnetic shaker previously described.[2]

The values of I_{norm} in Table S1 arise from the experimental spectra measured using the flip angles listed for the sample containing 5.2 mM [IrCl(COD)(d_{22}-IMes)] with 52

mM of ^{15}N labelled pyridine in methanol-d_4, where the hyperpolarisation step consisted of shaking for 10 s in a 5 mG magnetic field. The spectra taken with the parameters in Table S1 are displayed in Figure S5.

In order to exemplify that this sequence is robust to the exact parameters chosen, another set of parameters was chosen, optimised for 7 points this time over a 30 s observation window. The same sample and hyperpolarisation process were used as in the previous experiment. The chosen parameters are displayed in Table S2 and spectra are shown in Figure S6a. Figure S6 shows the consequence of choosing more points over a longer period of time on the overall signal by comparing a spectrum from Figures 1 and 2a.

n	t_n(s)	$M_{z,n}$	$M_{xy,n}$	ϑ_n (°)	$v_{p,n}$ (μs)	$I_{norm.}$ (%)
1	0	1.000	0.201	11.61	3.48	100.0
2	5	0.824	0.201	14.13	4.24	100.0
3	10	0.673	0.201	17.40	5.22	99.3
4	15	0.540	0.201	21.86	6.56	99.1
5	20	0.422	0.201	28.47	8.54	100.0
6	25	0.312	0.201	40.11	12.03	101.9
7	30	0.201	0.201	90.00	27.00	114.7
8	35	0.000	0.201	N/A	N/A	N/A

Table S2 – Calculated parameters for a sample containing 5.2 mM d_{22}-IMes with 52 mM of ^{15}N labelled pyridine in methanol-d_4, which has a measured T_1 of 29.0 s and d_{90} of 27 μs. All parameters were calculated from equations 1, 3 and 4 with the exception of the I_{norm} parameter, which is the normalised integral of the experimental data using the listed flip angles.

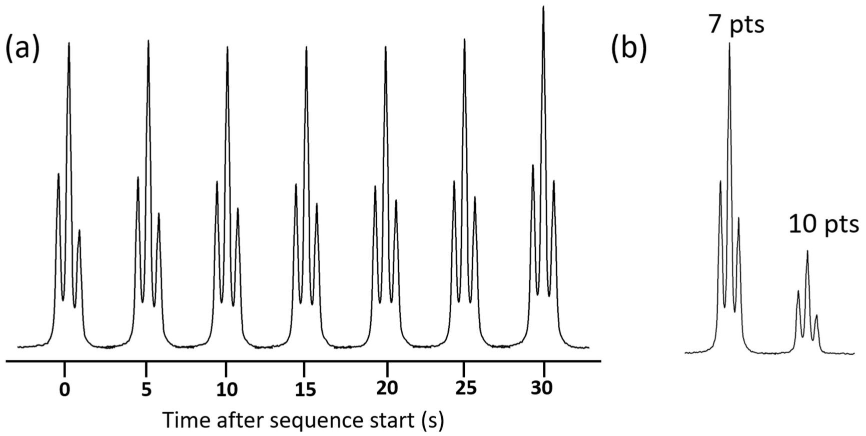

Figure S6 – (a) Spectra measured as a function of time measured after initial pulse, using the parameters listed in Table 2 for a sample containing 5.2 mM [IrCl(COD)(d_{22}-IMes)] with 52 mM of ^{15}N labelled pyridine in methanol-d_4. The sample was loaded with 4 Bar of *p*-H_2 (99%) and shaken for 10 s in a 5 mG magnetic field obtained using a magnetic shaker previously described.[4]

It is likely that the observed increase seen for the last data point when a hard 90° pulse is used is an artefact of the pulse duration being slightly off. However, this sequence has been shown to be stable and allow the monitoring of a chemical reaction by observing the change in intensity over the duration of the measurement. Thus we have assembled a rapid single-shot method of detecting chemical change, which has the potential to be used over a wide range of materials, reactions and NMR sensitive nuclei.

The data in Figure 4 of the manuscript was collected using this protocol. In this case, the T_1 was estimated to be 138.4 seconds and variable flip angle 13 measurements were conducted.

4 Dimerization of tris(*tert*-butoxy)silanol

When the reaction of tris(*tert*-butoxy)silanol is completed in the presence of a substoichiometric amount of triflic anhydride a new product peak is seen (δ_{Si} −93.2) in addition to the triflate product which has been characterised previously (δ_{Si} −102.7) as seen in Figure S7. We attribute this signal to the product of tris(*tert*-butoxy)silanol dimerization formed by subsequent reaction of tris(*tert*-butoxy)silanol with the triflate intermediate.

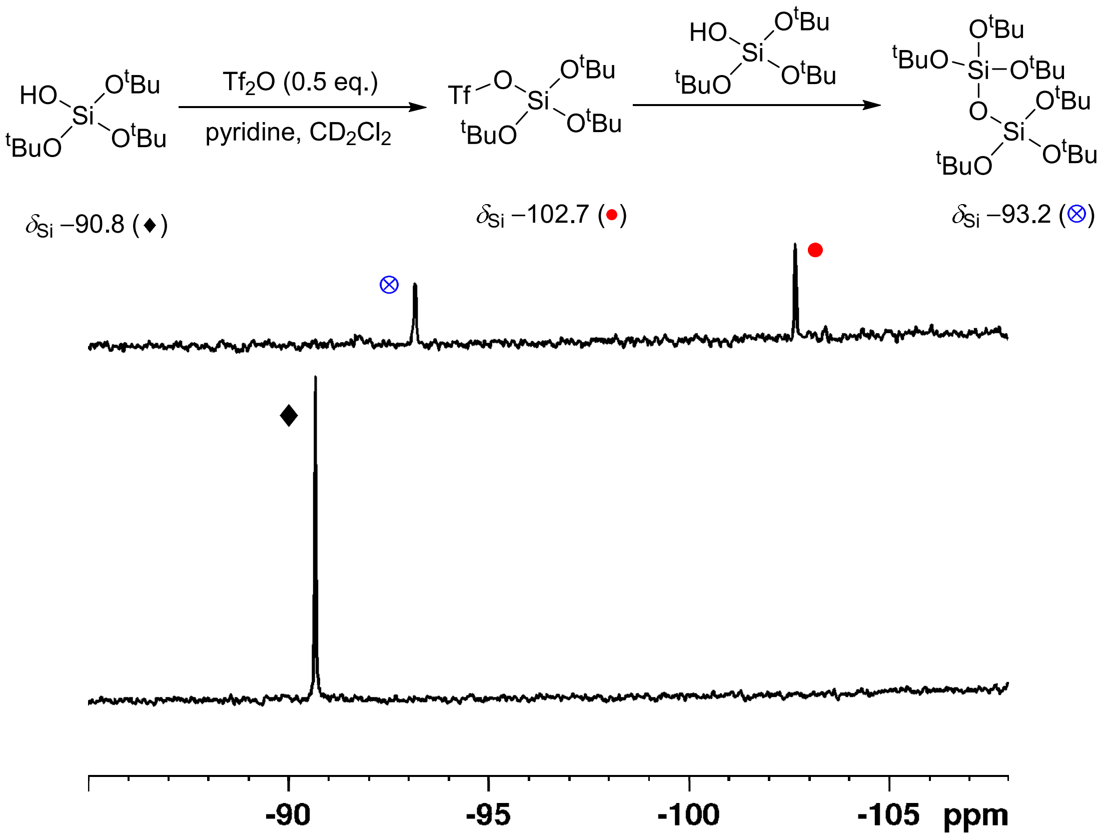

Figure S7 –Dimerization of tris(tert-butoxy)silanol. (top) Proposed reaction scheme. (bottom) ^{29}Si thermal NMR taken over 256 transients of the starting material and products after reaction at room temperature.

5 Calculation of Gibbs Free Energy

The ligand dissociation rate constants for equatorially bound $BnNH_2$ and hydride ligands were calculated using EXSY spectroscopy and fitted using previously reported methods.[5] These values were determined over a temperature range of 263-298 K. Subsequently the Gibbs Free Energy of the equilibrium ($\Delta G^{\neq}$) was determined using the linear form of the Eyring-Polanyi equation (Equation 5)

$$ln\frac{k}{T} = \frac{-\Delta H^{\neq}}{RT} + ln\frac{k_B}{h} + \frac{\Delta S^{\neq}}{R} \qquad (5)$$

A plot of $\frac{1}{T}$ vs $ln\frac{k}{T}$ was then used to determine the enthalpy (gradient, $-\frac{\Delta H^{\neq}}{R}$) and entropy (intercept, $ln\frac{k_B}{h} + \frac{\Delta S^{\neq}}{R}$) of the processes.

Table S3 summarizes the values of $\Delta G^{\neq 298}$ for $BnNH_2$ and hydride loss from the active SABRE complexes at 298 K.

Pre-Catalyst	$\Delta G^{\neq 298}$ $BnNH_2$ / $KJmol^{-1}$	$\Delta G^{\neq 298}$ Hydride/ $KJmol^{-1}$
**1**	66.794 ± 0.002	68.806 ± 0.001
**2**	66.164 ± 0.002	68.558 ± 0.000
**3**	64.931 ± 0.007	66.640 ± 0.007
**4**	63.337 ± 0.009	65.496 ± 0.009
**5**	62.539 ± 0.040	63.493 ± 0.040

6 References

[1] R. V. Shchepin, L. Jaigirdar, T. Theis, W. S. Warren, B. M. Goodson, E. Y. Chekmenev, *J. Phys. Chem. C* **2017**, *121*, 28425-28434.

[2] R. E. Mewis, K. D. Atkinson, M. J. Cowley, S. B. Duckett, G. G. R. Green, R. A. Green, L. A. R. Highton, D. Kilgour, L. S. Lloyd, J. A. B. Lohman, D. C. Williamson, *Magn. Reson. Chem.* **2014**, *52*, 358-369.

[3] O. Semenova, P. M. Richardson, A. J. Parrott, A. Nordon, M. E. Halse, S. B. Duckett, *Anal. Chem.* **2019**, *91*, 6695-6701.

[4] W. Iali, S. S. Roy, B. J. Tickner, F. Ahwal, A. J. Kennerley, S. B. Duckett, *Angew. Chem.* **2019**, *131*, 10377-10381.

[5] (a) C. Godard, J. Lopez-Serrano, M. D. Galvez-Lopez, M. Rosello-Merino, S. B. Duckett, I. Khazal, A. Lledos, A. C. Whitwood, *Magn. Reson. Chem.* **2008**, *46*, S107-S114; (b) P. J. Rayner, P. Norcott, K. M. Appleby, W. Iali, R. O. John, S. J. Hart, A. C. Whitwood, S. B. Duckett, *Nat. Commun.* **2018**, *9*, 4251.
